# Solution conformation and flexibility of capsular polysaccharides from *Neisseria meningitidis* and glycoconjugates with the tetanus toxoid protein

**DOI:** 10.1038/srep35588

**Published:** 2016-10-26

**Authors:** Ali Saber Abdelhameed, Gordon A. Morris, Fahad Almutairi, Gary G. Adams, Pierre Duvivier, Karel Conrath, Stephen E. Harding

**Affiliations:** 1 National Centre for Macromolecular Hydrodynamics, University of Nottingham, Sutton Bonington LE12 5RD, UK; 2 Department of Pharmaceutical Chemistry, College of Pharmacy, King Saud University, P.O. Box 2457, Riyadh, 11451, Kingdom of Saudi Arabia; 3 Department of Chemical Sciences, School of Applied Science, University of Huddersfield, Queensgate, Huddersfield, HD1 3DH, UK; 4 Biochemistry Department, Faculty of Science, University of Tabuk, P.O. Box 741-Tabuk 71491 Saudi Arabia; 5 Insulin and Diabetes Experimental Research (IDER) Group, University of Nottingham, Faculty of Medicine and Health Science, Clifton Boulevard, Nottingham NG7 2RD, UK; 6 GSK Vaccines, Rue de l’Institut 89, B1-330 Rixensart, Belgium

## Abstract

The structural integrity of meningococcal native, micro-fluidized and activated capsular polysaccharides and their glycoconjugates – in the form most relevant to their potential use as vaccines (dilute solution) - have been investigated with respect to their homogeneity, conformation and flexibility. Sedimentation velocity analysis showed that the polysaccharide size distributions were generally bimodal with some evidence for higher molar mass forms at higher concentration. Weight average molar masses *M*_w_ where lower for activated polysaccharides. Conjugation with tetanus toxoid protein however greatly increased the molar mass and polydispersity of the final conjugates. Glycoconjugates had an approximately unimodal log-normal but broad and large molar mass profiles, confirmed by sedimentation equilibrium “SEDFIT MSTAR” analysis. Conformation analysis using HYDFIT (which globally combines sedimentation and viscosity data), “Conformation Zoning” and Wales-van Holde approaches showed a high degree of flexibility – at least as great as the unconjugated polysaccharides, and very different from the tetanus toxoid (TT) protein used for the conjugation. As with the recently published finding for Hib-TT complexes, it is the carbohydrate component that dictates the solution behaviour of these glycoconjugates, although the lower intrinsic viscosities suggest some degree of compaction of the carbohydrate chains around the protein.

*Neisseria meningitidis*, is a non-motile Gram negative oval bacterium, occurring typically in pairs, with adjacent sides flattened or concave^1^,^2^. Meningococcus is highly adapted to the human nasopharynx (suggesting a long and intimate commensal relationship with man) as well as being highly transformable bacterium, capable of acquiring and integrating DNA from a range of microbes with which it comes into contact^3^. It is principally known for its role in meningitis and other forms of meningococcal disease such as meningococcemia. *N. meningitidis* is a major cause of morbidity and mortality during childhood in industrialized countries and is responsible for epidemics in Africa and in Asia^4^. A small minority of those who become infected eventually will develop an acute inflammation of the meninges “meningitis”.

*N. meningitidis* is an encapsulated bacterium, with many isolates enveloped by a polysaccharide capsule, a major antigenic structure that used to classify meningococcal isolates by immunological means into serogroups. Each serogroup corresponds to a chemically and antigenically distinct capsular polysaccharide and, although 13 distinct serogroups have been described^5^,^6^, virtually all isolates from invasive disease belong to one of six serogroups, namely: A, B, C, W-135, X and Y^7^,^8^. Serogroup A meningococci are the major cause of the large, cyclic epidemics in Africa and Asia, while in industrialized nations 30–70% of the disease is caused by serogroup B organisms. Serogroup C meningococci are particularly associated with, usually, smaller-scale outbreaks worldwide^7^,^8^,^9^. Serogroup X is more restricted to parts of sub-Saharan Africa^9^. Serogroup Y meningococci are currently accounting for over 30% of cases in the USA. Serogroup W-135 meningococci was associated with large outbreaks among pilgrims to the Hajj in Saudi Arabia in 2000 and was responsible for the epidemic in Burkina Faso in 2002^6^. The polysaccharide capsules of *N. meningitidis* are important determinants of virulence. Mutants without capsular expression are serum sensitive (*i.e.* killed by complement, and non-pathogenic). These polysaccharides are large, unbranched structures made up of O-acetylated residues in the 3 position of D-mannosamine-6 phosphate linked (1→6). These are O-acetylated repeating units of *N*-acetylmannosamine, linked with α-(1→6) phosphodiester bonds in case of serogroup A:


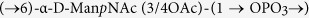


(Ac = acetyl) or in case of serogroup C these are linear polymers made up of partly O-acetylated repeating units of sialic acid, linked with α-(2→9) glycosidic bonds:


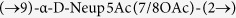


W-135 polysaccharide consists of partly O-acetylated alternating units of sialic acid and D-galactose, linked with α-(2→6) and α-(1→4) glycosidic bonds:


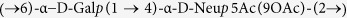


*Neisseria meningitidis* group Y polysaccharide consists of partly O-acetylated alternating units of sialic acid and D-glucose, linked with α-(2→6) and α-(1→4) glycosidic bonds:


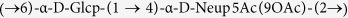


see e.g.^10^,^11^,^12^,^13^. The first successful capsular polysaccharide vaccines against groups A and C were developed in response to epidemics of meningitis among US military recruits^14^,^15^. However, it was found that the meningococcal polysaccharides proved to be poor immunogens in infants and fail to induce immunological memory in people of any age. Additionally, immune hypo-responsiveness is recorded after repeated vaccination with group C polysaccharide vaccine, which may cause difficulties for individuals who need long-term protection^16^. These disadvantages motivated extensive research to produce a polysaccharide-protein conjugate vaccine. Successes were subsequently achieved in the development of conjugate vaccines against *Haemophilus influenzae* type *b* (Hib) and *Streptococcus pneumoniae* thereby showing that the immunogenicity of polysaccharides could be improved by chemical conjugation to a protein carrier and eliciting a T-cell dependent anti-saccharide antibody response. The resulting polysaccharide – protein conjugate vaccines - are safe, immunogenic in young infants and induce long-term protection. In November 1999, meningococcal group C conjugate vaccine was introduced into routine immunisation in the UK^17^. Bivalent A plus C polysaccharide conjugate vaccines, have been assessed in clinical trials and were well tolerated and immunogenic in infants, toddlers, and adults^18^,^19^,^20^. Vaccine manufacturers have now developed conjugate vaccine combinations incorporating groups A, C, Y, and W-135 see e.g.^11^,^21^,^22^,^23^.

This present study investigates a recently FDA approved conjugate vaccine against serogroups A, C, W-135 and Y namely, Nimenrix® produced by Pfizer Ltd., UK. The focus of this study has been sedimentation velocity in the analytical ultracentrifuge, together with sedimentation equilibrium and viscosity to examine the molecular integrity of the samples in terms of heterogeneity, molar mass distribution and conformational flexibility of purified native, micro-fluidized and activated (chemically modified to facilitate conjugation) capsular polysaccharides from *N. meningitidis* serogroups as well as meningococcal-tetanus toxoid (TT) conjugates. It follows our recent studies on (i) *S. pneumoniae* polysaccharides^24^; (ii) the tetanus toxoid protein^25^ and (iii) on the Hib-TT system^26^ the latter study clearly demonstrating it was the carbohydrate polymer component rather than the protein which dictated the solution properties rather than the protein component for that glycoconjugate vaccine system. We now seek to establish if this is true also for the Men-TT system.

## Results

### Sedimentation coefficient distributions and molar mass

**Figure 1:**
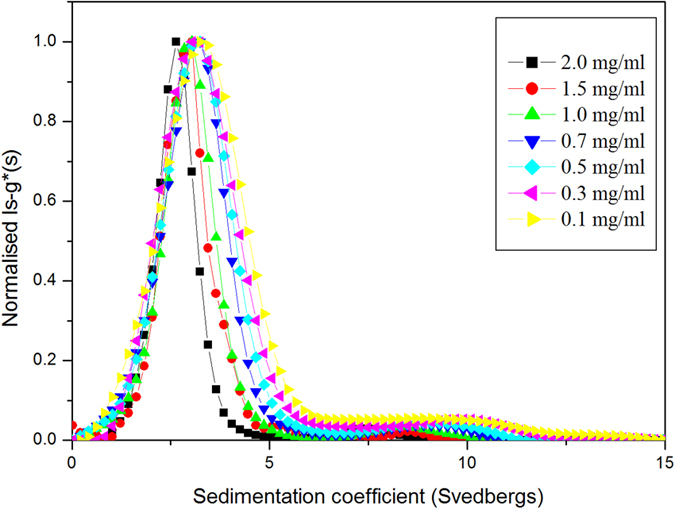
Sedimentation coefficient concentration distribution, least squares g*(*s*) vs *s* profile for ADH-activated *Men*A in phosphate-chloride buffer pH~6.8, I = 0.1, at 20.0 °C at loading concentration between 0.1–2.0 mg mL^−1^. Rotor speed = 45000 rpm. The plot has been normalized so that the main peak height is set to 1 for each concentration. The steady movement of the distribution to higher *s* (decreasing non-ideality) as the concentration is decreased can be clearly seen.

Analysis of the sedimentation coefficient distributions g*(s) vs *s* profiles in phosphate-chloride buffer (pH = 6.8, I = 0.10) showed that, in common with *Hib* polysaccharides^26^ the native *Men* polysaccharides are bimodal systems with the high molar mass components ranging from 5% by mass in native *Men*Y to 20% in native *Men*A (for both Na^+^ and Ca^2+^ salts). The high molar mass component was relatively less significant at low concentrations. The micro-fluidized (M-F) *Men* polysaccharides were characterized by lower sedimentation coefficients with the two components still present in M-F *Men*A. All other micro-fluidized *Men* polysaccharides appear to be mostly unimodal at different concentrations. The activated *Men*A and *Men*C polysaccharides were very nearly unimodal (Fig. 1). The bimodal structure of the polysaccharide distributions - particularly the native *Men* polysaccharides - are considered to be due to a higher degree of polymerization particularly for *Men*A Na^+^ and Ca^2+^ salts - see for example^11^. The g*(s) plots for all samples also showed the classical increase in the value of sedimentation coefficient *s* with the decrease in concentration, *c*, due to lowering non-ideality effects which vanish as *c*→0. After normalisation of *s* values measured in the buffer to standard solvent conditions (the viscosity and density of water at 20.0 °C) to give *s*_20,w_, standard reciprocal plots 1/*s*_20,w_ vs *c* were used to obtain the non-ideality free *s*°_20,w_ and concentration dependence Gralen values *k*_s_ and are shown for comparison in Table 1.

**Figure 2:**
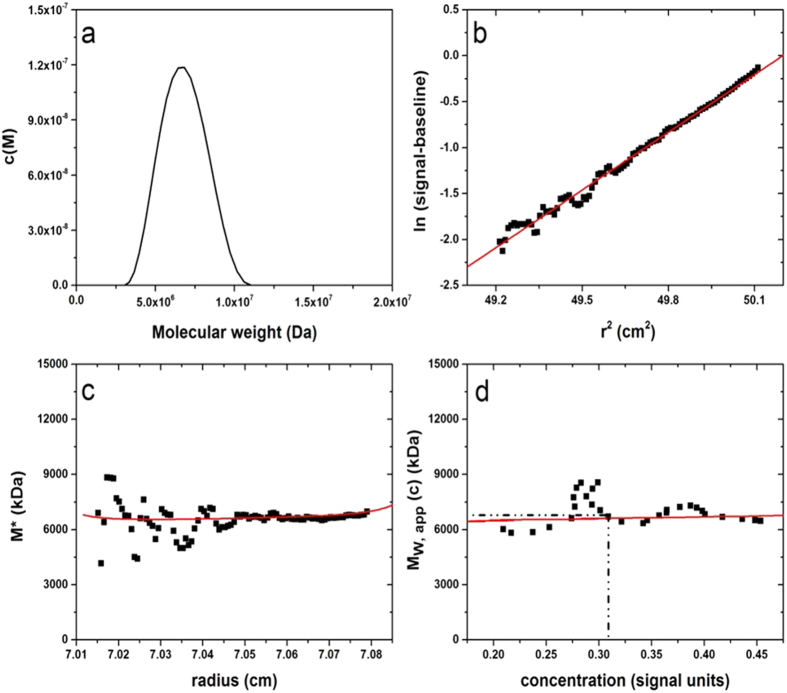
*SEDFIT-MSTAR* output for analysis of *Men*A with spacer conjugate in phosphate-chloride buffer pH~ 6.8, I = 0.1, at 20.0 °C and at a loading concentration of 0.3 mg mL^−1^. Rotor speed = 2000 rpm. (**a**) low resolution molar mass distribution; (**b**) log concentration versus the square of the radial displacement from the centre of rotation. (**c**) extrapolation of the M* function to the cell base to yield the “whole distribution” apparent weight average molar mass *M*_*w,app*_ = (7500 ± 540) kDa; (**d**) plot of the point average molar mass (local molar mass) – obtained by taking the derivative of the data from plot (**b**) versus local concentration *c*(*r*) in the analytical ultracentrifuge cell.

Sedimentation equilibrium was then used to determine average molar masses using the *SEDFIT-MSTAR* and *MFIT* algorithms which give weight-average molar masses and z-average molar masses, respectively for the prime parameters (Table 1, see also Fig. 2). With the glycoconjugates generally a very low rotor speed was required to register a measurable distribution at equilibrium. In the case of the *Men* A conjugates without spacer it was not possible to get a measurable distribution, so molar masses were obtained from comparison of the sedimentation coefficient values with *Men* A conjugates with spacer and MHKS power law coefficients *b* of 0.4 and 0.5, in the relation *s* ~ *M*^b^, a range consistent with a macromolecule with a high degree of flexibility (see the consideration of conformation and flexibility below).

**Figure 3:**
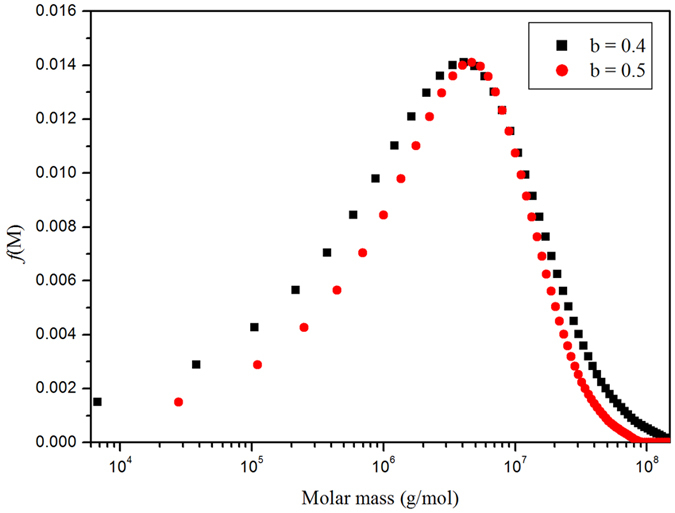
*f*(*M*) distribution profile (molar mass axis on a logarithmic scale) from Extended Fujita analysis of the sedimentation velocity data for the MenC conjugate with spacer. Distributions for 2 plausible values for the sedimenation power law coefficient *b* are shown.

Sedimentation coefficient distributions could then be transformed into distributions of molar masses for the Men-TT conjugates using the *Extended Fujita Approach* of Harding, *et al*.^27^ (Fig. 3)

The transformation is as follows:


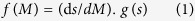


with


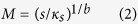


and


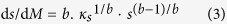


*b* as referred to above is a conformation parameter that has already been estimated for number of polysaccharides in particular solvent conditions^27^ and *κ*_*s*_ can be found from equation (2) provided that at least one value of *M* (*e.g.* the weight average over the whole distribution, *M*_w_ from sedimentation equilibrium) is known for one value of *s* (*e.g.* the weight average *s* value).

### Intrinsic viscosity

Intrinsic viscosity values resulting from three different extrapolation methods (due to Huggins, Kraemer and Solomon & Ciuta) are reported in Table 2. Consistent with the behaviour of the sedimentation coefficients, the intrinsic viscosity results for the polysaccharides are decreasing with the size reduction and activation of native polysaccharide. Intriguingly they are generally lower for the glycoconjugates even though the sedimentation coefficients and molar masses are much higher, although still an order of magnitude higher than for compact globular particles (including the tetanus toxoid protein^25^,^26^ and large molar mass spherical viruses^28^). Nonetheless they are lower than for the *Hib-*glycoconjugates^26^ indicating greater compaction.

## Discussion

Based on the hydrodynamic data we can make some clear inferences about the conformation of the glycoconjugate

### Wales-van Holde ratio

The Wales-van Holde ratio^29^, *R* *=* *k*_*s*_*/[η]* is perhaps the simplest guidance/indicator of a molecule conformational flexibility. The limits are ~1.6 for a compact sphere or a non-draining random coil, and ~0.1 for a stiff rod^30^. From Table 2, it can be seen that some of the purified native, micro-fluidized and activated capsular polysaccharides from *N. meningitidis* and the final conjugates have Wales-van Holde ratios corresponding to flexible random coil structures. Native MenA-Na^+^, native MenY, M-F (microfluidized) MenC, M-F Men W135, M-F MenY, activated MenA, MenA-Ca^2+^ salt without spacer, MenC conjugate with spacer, MenC conjugate without spacer and MenY conjugate without spacer are showing more flexible structures according to their high *R* values.

### Translational frictional ratio

The translational frictional ratio, *f/f*_*o*_ is a parameter which depends on conformation *and* molecular expansion through hydration effects^31^. It can be measured experimentally from the sedimentation coefficient and molar mass:


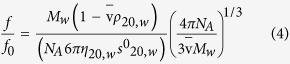


where N_A_ is Avogadro’s number, *f* is the friction coefficient of the molecule and *f*_*o*_ the corresponding value for a spherical particle of the same mass and (anhydrous) volume^31^. Values of *f/f*_*o*_ increase with increasing chain stiffness, although they are also molar mass dependent. The values for the frictional ratio in Table 2 are also consistent with a flexible coil structure.

### Persistence length L_p_

For a more quantitative estimate of chain flexibility we can use the persistence length *L*_*p*_, which has theoretical limits of 0 for a random coil and ∞ for a stiff rod. Practically the limits are ∼1–2 nm for a random coil and ∼200–300 nm for a very stiff rod shaped macromolecule. Several methods are available for estimation of *L*_*p*_ using either intrinsic viscosity^32^,^33^ or sedimentation coefficient^34^,^35^ measurements. For example the relation^32^,^33^.


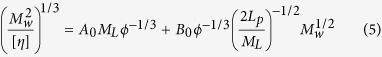


where *ϕ* is the Flory-Fox coefficient (2.86 × 10^23^ mol^−1^) and A_0_ and B_0_ are tabulated coefficients, and the Yamakawa–Fujii equation^34^.


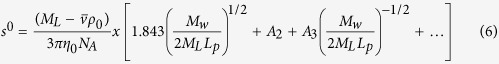


**Figure 4:**
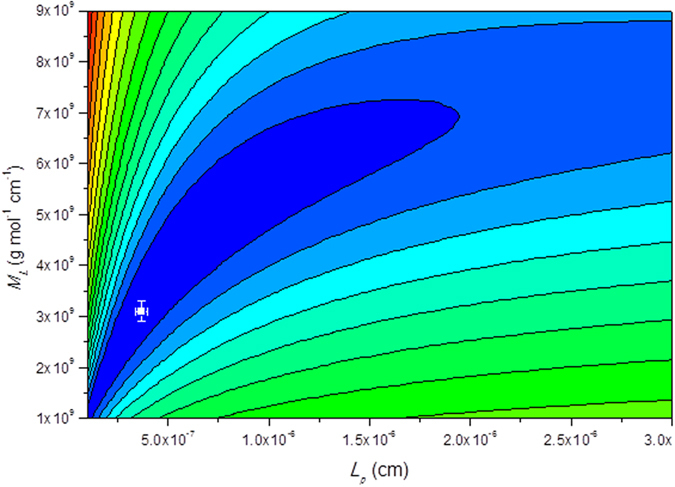
‘HYDFIT’ contour plots of mass per unit length *M*_*L*_ versus persistence length *L*_*p*_ for M-F MenY. The contours of different colour correspond to different values of a target function: the minimum value (indicated by the cross) corresponds to the best fit. The plot yields *L*_*p*_ ~ 3.7 (nm) and *M*_*L*_ ~ 3.1 × 10^9^ (g mol^−1^ cm^−1^) at the minimum target (error) function value of 0.05.

Yamakawa and Fujii showed that A_2_ can be considered as –*ln(d/2L*_*p*_) and A_3_ = 0.1382 if the *L*_*p*_ is much higher than the chain diameter, *d*. Difficulties arise if the mass per unit length is not known, although both relations have now been built into an algorithm Multi-HYDFIT^35^ which estimates the best estimates or best range of values of *L*_*p*_ and *M*_*L*_ based on minimization of a target function *Δ*. An estimate for the chain diameter *d* is also required but extensive simulations have shown that the results returned for *L*_*p*_ are relatively insensitive to the value chosen for *d* which was fixed at an average of ∼0.8 nm see e.g.^36^,^37^ *M*_*L*_ and *L*_*p*_ were treated as variables and the minimum value of the target function *Δ* was estimated on a 2D contour plot for each sample: an example is given in Fig. 4, and the values estimated for Men samples given in Table 3. All the values are consistent with flexible random coil structures, including the polysaccharides both prior and after conjugation.

These results are in support of suggesting highly flexible random coil structures. Molecular flexibility is increasing by size reduction and activation and more because of conjugation. The estimation for the mass per unit lengths within experimental errors are in good agreement with the predicted values from the repeating unit structures.

### Sedimentation Conformation Zoning

Assignment of the conformation type or “zone”^38^,^39^ utilizes persistence length *L*_*p*_ and mass per unit length *M*_*L*_ parameters via plotting *k*_*s*_*M*_*L*_ versus [*s*]/*M*_*L*_, where [*s*] is given by:


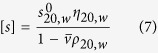


**Figure 5:**
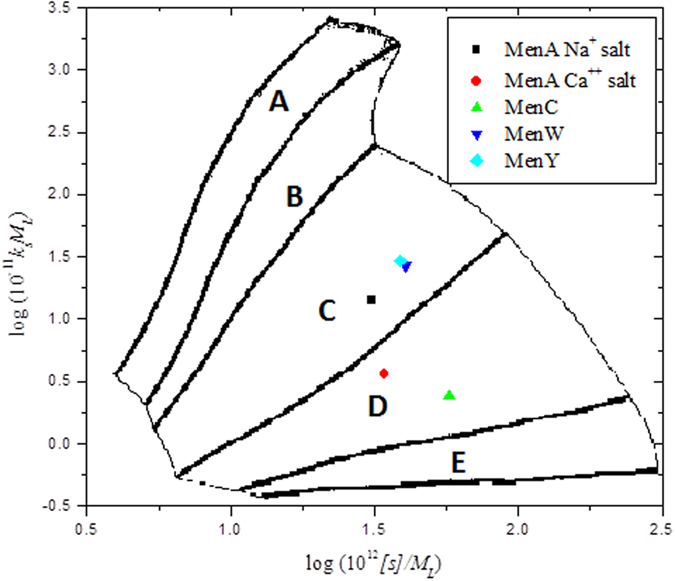
Conformation zoning plots of meningococcal native polysaccharides, showing either semi-flexible (Zone C) or highly flexible random coil structures (Zone D). The other zones^38^,^39^ are A: rigid rod; B: rod; E: globular or branched

**Figure 6:**
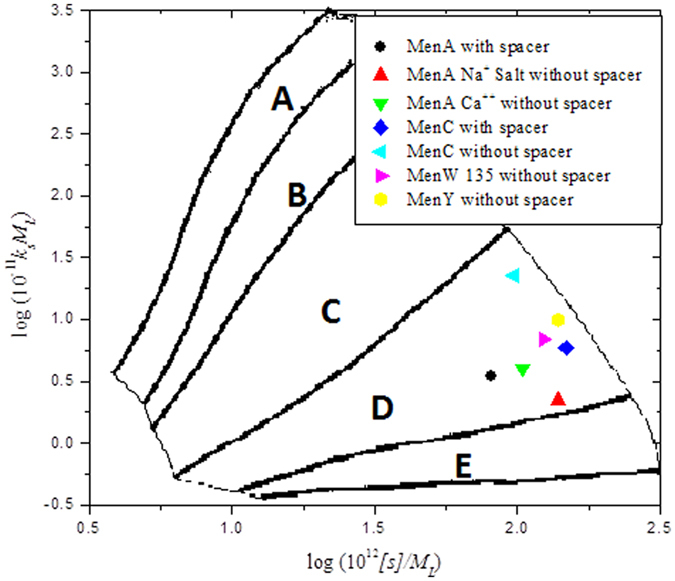
As Fig. 5, but for meningococcal conjugates, showing highly flexible random coil structures (Zone D).

A Conformation Zoning plot for native polysaccharides (Fig. 5) shows semi-flexible structures for native MenA in its Na^+^ salt which becomes more flexible in the Ca^2+^ salt. It also shows semi-flexible structures of native MenW135 and native MenY and more flexible random coil structure of native MenC. After the size reduction process “micro-fluidization” and the activation of the polysaccharides they are shown to be adopting semi-flexible structures. On the other hand, the zoning plot of meningococcal conjugates (Fig. 6) confirms the significant increase in the flexibility of the conjugates (Zone D – random coils) compared to their native, micro-fluidized and activated counterparts.

## Concluding Remarks

The conformation analyses suggest semi-flexible random coil structures for MenA Na^+^ salt, MenW135 and MenY polysaccharides and more flexible random coil structures for MenA Ca^2+^ salt and MenC polysaccharides. The micro-fluidization significantly reduced the viscosity and molar mass. Conformation of those micro-fluidized polysaccharides has changed to less flexible structures. Similarly the conformation of the activated polysaccharides slightly changed to less semi-flexible structures, their molar masses have also decreased from the native polysaccharides. The conjugation process has altered the viscosity of the final conjugates and remarkably increased the molar masses of the final conjugates. However, the conformation of the conjugates has become more flexible than native, micro-fluidized and activated polysaccharides and that the conjugates are highly flexible chains or highly flexible random coil structures. The lower intrinsic viscosities compared with the native or activated polysaccharides is indicative of some degree of wrapping of the polysaccharide chains around the tetanus toxoid protein core, although, like with glyco-vaccines based on *Hib*^26^ it seems it is the carbohydrate polymer component which dictates the hydrodynamic properties of these substances.

## Methods

### Sample preparation

Preparation of polysaccharides was similar to that described for *Streptococcus pneumoniae*^24^. Prior to their conjugation, polysaccharides undergo size reduction by micro-fluidization in a high pressure homogenizer, which significantly reduces the viscosity and is likely to enhance the polysaccharides reactivity during conjugation^22^,^40^,^41^. During the development of the quadrivalent conjugate vaccine used in this study, different conjugation chemistries have been used in coupling of the polysaccharides to the carrier protein using a spacer or direct linking them without a spacer. Hence, samples used in this study are native polysaccharides from serogroups C (MenC), W-135 (MenW135), Y (MenY) and serogroup A sodium salt (MenA-Na^+^) and calcium salt (MenA-Ca^2+^). Micro-fluidized polysaccharides from serogroups A, C, W-135 and Y, activated polysaccharides from serogroups A and C, were also examined. Serogroup A conjugate without spacer (MenA without spacer), sodium salt conjugate with spacer (MenA-Na^+^ with spacer), calcium salt conjugate with spacer (MenA-Ca^2+^ with spacer) and serogroup C conjugate with spacer (MenC with spacer), conjugate without spacer (MenC without spacer) and serogroup W-135 conjugate without spacer (MenW135 without spacer) and serogroup Y conjugate without spacer (MenY without spacer), were also investigated.

### Polysaccharide-protein conjugation with a spacer

The covalent binding of the polysaccharides and the spacer adipic acid dihydrazide (ADH) is carried out using a coupling chemistry by which the polysaccharides are activated under controlled conditions by a suitable cyanylating agent, 1-cyano-4-dimethylamino-pyridinium tetrafluoroborate (CDAP) see e.g.^41^,^42^. CDAP reacts with the polysaccharide, exchanging a cyano group for hydroxyl hydrogen, hydroxyl groups being abundant on the polysaccharide, creating a highly reactive cyanoester. The activation is best done at pH 9–10, and in fact there is a strong pH dependence on CDAP polysaccharide (PS) activation efficiency^40^. The spacer reacts with the cyanylated-polysaccharide through its hydrazino groups, to form a stable isourea link between the spacer and the polysaccharide. The conjugation of the tetanus toxoid (TT) with PS-ADH consists of the covalent binding of the protein on the derivatized PS using 1-ethyl-3-(3-dimethylaminopropyl) carbodiimide (EDC)^43^,^44^. A stable peptidic link is formed between the carboxylic group of the protein and the hydrazino group of the spacer (ADH).

### Conjugation without a spacer

As an alternative to using a linker (spacer), direct linkage can be used. Activation of polysaccharides with CDAP introduces a cyano group in the polysaccharides and dimethylaminopyridine (DMAP) is liberated. The cyano group reacts with NH_2_-groups of the protein during the subsequent coupling phase leading to binding of the polysaccharide to the TT protein by a way of an isourea link.

Prior to their purification, the conjugates are filtered through a 10 μm membrane in order to remove potential aggregates. The conjugates are then purified on a Sephacryl S400HR column to remove the by-products and unbound protein and/or polysaccharides. They are finally sterile filtered on a 0.22 μm membrane. All samples were dissolved in phosphate-chloride buffer pH~ 6.8, I = 0.1^45^, at 20.0 °C. All solutions were then diluted to the appropriate concentrations required for the hydrodynamic characterisations.

### Sedimentation velocity in the analytical ultracentrifuge

Sedimentation velocity experiments were performed using a Beckman (Palo Alto, CA, USA) Optima XL-I analytical ultracentrifuge equipped with Rayleigh interference optics and an automatic on-line data capture system. Conventional 12.0 mm double-sector epoxy cells with sapphire windows were loaded with 400 μL of different concentrations (0.1–2.0 mg mL^−1^) of each sample and a matching amount of the corresponding reference buffer in appropriate channels. Native, micro-fluidized and activated capsular polysaccharides from *N. meningitidis* were centrifuged at 45000 rpm at a temperature of 20.0 °C, while the glycoconjugates solutions were spun at 7000 rpm and the same temperature. Concentration profiles in the analytical ultracentrifuge cell were recorded using the Rayleigh interference optical system and converted to concentration (in units of fringe displacement relative to the meniscus, *j*) versus radial position, *r* at particular times *t*^46^. Data were analysed using the least squares boundary modelling referred to as the least squares ls-g*(s) model incorporated into the SEDFIT algorithm^47^. SEDFIT generates an apparent distribution of sedimentation coefficients in the form of g*(s) versus *s*_*T,b*_, where the (*) indicates that the distribution of sedimentation coefficients has not been corrected for diffusion effects, see e.g.^46^. The correction procedure requires assumptions about the friction coefficient not valid for continuous distributions of molecular weight/sedimentation coefficient, although for large polysaccharides/glycoconjugates, these corrections will be small^46^. This was followed by the correction to standard solvent conditions-namely the density and viscosity of water at 20.0 °C - to yield *s*_*20,W*_ using the utility program SEDNTERP^48^. Note that to account for non-ideality (co-exclusion and backflow effects), the apparent sedimentation coefficient (*s*_*20,w*_) was calculated at a series of different cell loading concentration and extrapolated to infinite dilution using the Gralén relation^49^:


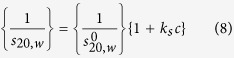


where *k*_*s*_ is the Gralén or concentration dependence coefficient.

### Sedimentation equilibrium (SE) in the analytical ultracentrifuge

Samples were prepared at a concentration of 0.3 mg/mL sufficiently low to minimize the effects of thermodynamic non-ideality. A volume of 1.0 ml of each sample was dialysed for 48 hours at the ambient temperature, each against 300 mL of the phosphate-chloride buffer. Sedimentation equilibrium experiments were also performed using the Beckman (Palo Alto, CA, USA) Optima XL-I analytical ultracentrifuge equipped with Rayleigh interference optics and an automatic on-line data capture system. The modified long (20.0 mm) optical path length double-sector titanium cells with sapphire windows were loaded with 0.15 mL of dialysed sample and a matching amount of reference buffer dialysate in appropriate channels. Samples were centrifuged at rotor speeds selected to give a sufficient fringe increment from meniscus to base^50^ *i.e.* 4000, 9000, 9000 and 2000 rpm for native, micro-fluidized, activated polysaccharides and glycoconjugates, respectively. Using the Rayleigh interference optical system, scans were taken every hour and equilibrium was reached after approximately 48 hours. Record of the relative concentration distribution of the solute at equilibrium was analysed to give the weight average apparent molar mass *M*_*w,app*_ using the *SEDFIT-MSTAR* algorithm^51^ based on an earlier algorithm of Cölfen and Harding^52^ and the M* function of Creeth and Harding^53^. The use of the long path length cells (20.0 mm) meant that low loading concentrations can be used to give a sufficient signal (~0.3 mg mL^−1^). At such low concentrations, non-ideality effects will be small and hence the apparent weight average molar mass will be approximately equal to the true weight average molar mass *M*_*w*_. *M*_w_ from *SEDFIT-MSTAR* could then be combined with the weight average sedimentation coefficient to yield the molecular weight distribution^54^. We also estimate *M*_*z*_ using the *MFIT* algorithm of Ang and Rowe^55^.

### Viscometry

Dynamic viscosity measurements for *Men* native, *Men*-ADH capsular polysaccharides and the *Men*-TT conjugate, were carried out using the automated micro-viscometer Anton Parr AMVn (Anton Parr, Graz, Austria) across a concentration series from 0.1–2.0 mg mL^−1^. The rolling ball viscosity method measures the time of a steel ball needed to roll in the 1.6 mm diameter silanized glass capillary containing each sample. The experiment was performed at different reclining angles of 70° (*n* = 4 times), 60° (*n* = 4 times) and 50° (*n* = 6 times) under precise temperature control (20.00 ± 0.01) °C. Huggins^56^ and Kraemer^57^ extrapolations forms were performed to obtain the intrinsic viscosity, as described in ref 26. Intrinsic viscosities were also estimated using the Solomon – Ciutâ relation which represents a combination of the opposite trends of the Huggins and Kraemer relations allowing estimation of [*η*] without extrapolation^58^,^59^: Nonetheless as a check on consistency of data such estimates are still registered at different concentrations.

## Additional Information

**How to cite this article**: Abdelhameed, A. S. *et al*. Solution conformation and flexibility of capsular polysaccharides from *Neisseria meningitidis* and glycoconjugates with the tetanus toxoid protein. *Sci. Rep.* **6**, 35588; doi: 10.1038/srep35588 (2016).

**Publisher’s note:** Springer Nature remains neutral with regard to jurisdictional claims in published maps and institutional affiliations.

## Figures and Tables

**Table 1:** Hydrodynamic properties of capsular polysaccharides and TT-glycoconjugates from *N. meningitides.*

Sample	*s*^*o*^_20,w_ (S)	*k*_*s*_ (mL g^−1^)	*M*_*w*_^*a*^(kDa)	*M*_*z*_^b^ (kDa)
Native polysaccharides				
*Men*A Na^+^	6.4 ± 0.3	250 ± 50	710 ± 35	775 ± 40
*Men*A Ca^2+^	4.5 ± 0.2	100 ± 40	620 ± 30	700 ± 35
*Men*C	6.2 ± 0.2	165 ± 35	1950 ± 100	1900 ± 95
*Men*W135	9.8 ± 1.2	400 ± 120	1350 ± 70	1400 ± 70
*Men*Y	8.8 ± 0.7	470 ± 80	1370 ± 70	1630 ± 80
M-F *Men*A	5.4 ± 0.2	195 ± 40	195 ± 10	240 ± 5
M-F *Men*C	4.0 ± 0.2	190 ± 40	185 ± 10	170 ± 5
M-F *Men*W135	2.7 ± 0.1	135 ± 20	275 ± 15	280 ± 20
M-F *Men*Y	2.4 ± 0.1	115 ± 10	110 ± 5	120 ± 5
Activated polysaccharides				
*Men*A-ADH	4.0 ± 0.1	170 ± 30	275 ± 15	350 ± 20
*Men*C-ADH	4.3 ± 0.1	85 ± 25	220 ± 10	245 ± 5
Glycoconjugates				
*Men*A with spacer	40 ± 1	25 ± 10	7900 ± 390	8000 ± 400
*Men*A-Na^+^ salt without spacer	44 ± 1	25 ± 5	10000^c^ 9600^d^	10100^c^ 9700^d^
*Men*A-Ca^2+^ salt without spacer	33 ± 1	45 ± 15	4900^c^ 5400^d^	4950^c^ 5450^d^
*Men*C with spacer	56 ± 2	55 ± 30	9500 ± 450	9800 ± 500
*Men*C without spacer	31 ± 1	250 ± 65	7800 ± 350	7900 ± 360
*Men*W135 without spacer	38 ± 1	80 ± 25	9800 ± 500	10300 ± 510
*Men*Y without spacer	44 ± 2	110 ± 90	10400 ± 450	10600 ± 480

^a^Sedimentation equilibrium *SEDFIT-MSTAR* analysis.

^b^Sedimentation equilibrium *MFIT* analysis.

^c^From comparison of the *s*^*o*^_*20,w*_ values with that of *Men*A with spacer and

assuming an MHKS *b* value = 0.4.

^d^From comparison of the *s*^*o*^_*20,w*_ values with that of *Men*A with spacer and assuming an MHKS *b* value = 0.5.

The standard errors quoted in this Table (and Tables 2 & 3) are due to the respective fits, taking into account other parameters such as error in the partial specific volume.

**Table 2:** Intrinsic viscosity [*η*], Wales-van Holde ratio *k*_*s*_ */[η]*and frictional ratios *f/f*_*0*_.

Sample	Huggins [*η*] (mL g^−1^)	Kraemer [*η*] (mL g^−1^)	Solomon-Ciuta [*η*] (mL g^−1^)	*k*_*s*_*/[η]*	*f/f*_*0*_
Native polysaccharides					
*Men*A Na^+^	170 ± 10	170 ± 10	170 ± 5	1.4	6
*Men*A Ca^2+^	115 ± 15	117 ± 15	117 ± 15	0.8	8
*Men*C	290 ± 5	284 ± 5	290 ± 5	0.6	13
*Men*W135	530 ± 10	510 ± 5	520 ± 5	0.8	7
*Men*Y	415 ± 5	410 ± 5	410 ± 5	1.1	7
M-F *Men*A	270 ± 10	280 ± 10	275 ± 10	0.7	3
M-F *Men*C	120 ± 10	120 ± 5	120 ± 5	1.6	4
M-F *Men*W135	100 ± 10	100 ± 10	100 ± 5	1.4	8
M-F *Men*Y	110 ± 5	105 ± 5	105 ± 5	1.1	6
Activated polysaccharides					
*Men*A-ADH	100 ± 5	105 ± 5	105 ± 5	1.6	6
*Men*C-ADH	115 ± 5	115 ± 5	115 ± 5	0.7	4
Glycoconjugates					
*Men*A with spacer	30 ± 2	29 ± 2	30 ± 2	0.8	5
*Men*A-Na^+^ salt without spacer	34 ± 3	34 ± 2	34 ± 2	0.8	*-*
*Men*A-Ca^2+^ salt without spacer	29 ± 1	28 ± 1	29 ± 1	1.5	*-*
*Men*C with spacer	46 ± 2	46 ± 2	46 ± 2	1.2	4
*Men*C without spacer	170 ± 15	170 ± 15	170 ± 15	1.5	7
*Men*W135 without spacer	125 ± 5	125 ± 5	125 ± 5	0.6	6
*Men*Y without spacer	89 ± 2	88 ± 2	88 ± 3	1.3	6

**Table 3:** Mass per unit length *M*_*L*_ and chain flexibility (*L*_*p*_) estimations from combining sedimentation and viscosity data through *HYDFIT.*

Sample	M_L_ (g mol^−1^ nm^−1^)	L_p_(nm)
Native polysaccharides		
*Men*A Na^+^	570 ± 20	5.9 ± 0.5
*Men*A Ca^2+^	365 ± 15	6.2 ± 0.4
*Men*C	325 ± 40	9.1 ± 0.7
*Men*W135	670 ± 30	6.8 ± 0.5
*Men*Y	630 ± 20	7.1 ± 0.4
M-F *Men*A	880 ± 20	13.5 ± 1.5
M-F *Men*C	485 ± 20	4.1 ± 0.5
M-F *Men*W135	220 ± 15	4.2 ± 0.4
M-F *Men*Y	310 ± 20	3.7 ± 0.3
Activated polysaccharides		
*Men*A-ADH	445 ± 15	4.5 ± 0.4
*Men*C-ADH	450 ± 20	3.6 ± 0.2
Glycoconjugates		
*Men*A with spacer	1400 ± 70	3.6 ± 0.3
*Men*A-Na^+^ salt without spacer	890 ± 60	6.5 ± 0.4
*Men*A-Ca^2+^ salt without spacer	890 ± 50	8.8 ± 0.6
*Men*C with spacer	1070 ± 50	1.8 ± 0.1
*Men*C without spacer	900 ± 20	3.9 ± 0.2
*Men*W135 without spacer	870 ± 20	3.0 ± 0.3
*Men*Y without spacer	900 ± 40	1.3 ± 0.4
